# Childhood cancer patients' experiences of a structured exercise program. A qualitative study using reflexive thematic analysis

**DOI:** 10.3389/fped.2025.1547822

**Published:** 2025-03-26

**Authors:** Dennis Wilke, Norbert W. Paul, Elias Dreismickenbecker, Francesca Alt, Marie A. Neu, Jörg Faber

**Affiliations:** ^1^ Institute for the History, Philosophy and Ethics of Medicine, University Medical Center of the Johannes Gutenberg-University Mainz, Mainz, Germany; ^2^ Department of Pediatric Hematology, Oncology, and Hemostaseology, Center for Pediatric and Adolescent Medicine, University Medical Center of the Johannes Gutenberg-University Mainz, Mainz, Germany; ^3^ Childhood Cancer Center Mainz, University Cancer Center Mainz (UCT Mainz), University Medical Center of the Johannes Gutenberg-University Mainz, Mainz, Germany

**Keywords:** childhood cancer, exercise therapy, pediatric exercise oncology, physical activity, qualitative study, reflexive thematic analysis

## Abstract

**Introduction:**

Undergoing cancer treatment as a child, adolescent or young adult involves decreased physical activity and fitness, which may further compromise the bodily and psychosocial well-being of young patients. As an element of supportive care, exercise interventions may counteract these adverse effects. However, knowledge about the impact of such interventions, and of patients' and parents' experiences of participation, is only beginning to emerge.

**Objectives:**

To explore patients' and parental experiences of participating in a structured, individualized exercise program lasting 8–10 weeks during intensive treatment in a German childhood cancer center as part of the clinical trial *FORTEe – Get strong to fight childhood cancer*.

**Methods:**

We conducted open qualitative interviews (*n* = 11) with children and adolescents (*n* = 6) and parents (*n* = 5), as well as participant observation during exercise sessions (*n* = 10). We used reflexive thematic analysis, as developed by Braun and Clarke, as our method of analysis.

**Results:**

We generated three themes: 1. *Feeling better in my body and experiencing my physical capabilities*; 2. *Gaining distance from illness and treatment*; and 3. *Being recognized and involved as a vulnerable and individual patient*. Moreover, we gained insights, regarding the burdensome impact of childhood cancer, and limitations of the exercise intervention. Participants reported almost exclusively positive experiences of participating in the program, which yielded benefits for the patients' physical fitness and capabilities, their bodily and psychological well-being and everyday life situation.

**Conclusion:**

This study supports the suitability and importance of making exercise therapy an integral element of supportive care for childhood cancer patients. Its insights may be helpful for health care professionals who plan on implementing, or who are involved in providing exercise interventions, as well as for scientists who accompany such interventions with qualitative research.

## 1 Introduction

Cancer treatment in children, adolescents and young adults is accompanied by burdensome side-effects as well as by decreased mobility and physical activity (1–3). The latter can have additional adverse effects such as impaired cardiorespiratory fitness (4), muscle strength and quality of life (5, 6), which may lead to a “downward spiral” of inactivity (7). Thus, adverse late effects of childhood cancer may be exacerbated (8).

In recent years, researchers have increasingly examined the effects of physical activity and exercising on the health and well-being of childhood cancer patients, establishing the field of *pediatric exercise oncology*. While evidence is still emerging, studies have shown that physical activity may be beneficial for childhood cancer patients, with regard to endpoints such as functional mobility, strength, cancer-related fatigue, or quality of life, and that exercise interventions are appreciated by patients (9–17). In their qualitative study, Götte et al. (18) found for example that patients reported predominantly positive attitudes towards physical activities during treatment. However, knowledge gaps regarding the effects and limitations of exercise interventions remain, and authors have called for larger (multisite) studies to be conducted to enhance evidence (19–21).

The International Pediatric Oncology Exercise Guidelines (iPOEG) (22) recommend that exercise prescription be guided by professionals with expertise in both cancer and exercise. To ensure safety and effectiveness, qualified staff are crucial to delivering exercise interventions appropriately and maintaining clear communication with patients and families, including assessing their preferences and potential barriers. In addition, Wurz et al. (20) have argued for greater adoption of patient-oriented research approaches that center and “activate the voices of end users”, as well as for the use of “alternative” and “varied” study designs in pediatric exercise oncology, and other researchers from this field have recommended “[…] physical exercise interventions be delivered according to the preferences and wishes of children and adolescents.” [(23); see also (14, 24)]. By drawing on the subjective perspectives and lived experiences of patients or families, qualitative approaches may contribute precisely to these recommendations. Qualitative methods are established both in research with childhood cancer patients (25), as well as in (health-related) physical activity and exercise research (26, 27), and authors have emphasized the relevance of qualitative methodology for the latter (28).

However, qualitative insights on childhood cancer patients' perspectives and experiences regarding exercise interventions are still rare. Using semi-structured interviews and grounded theory analysis, Götte et al. (18) have examined patients' attitudes to physical activity and to an exercise program during cancer treatment. While some barriers were identified, patients were motivated to participate in the exercise program and reported positive effects on physical and mental well-being. Grimshaw et al. (7) have conducted semi-structured interviews with parents to explore reasons for, and effects of physical inactivity during acute treatment, identifying the aforementioned “spiral” of inactivity and highlighting the need for support by the oncology team. Thorsteinsson et al. (29) have interviewed childhood cancer patients about their motivations to participate in a physical activity program during treatment. As part of the same overarching project, Petersen et al. (30) have examined the participation experiences of childhood cancer survivors and their parents, as well as their perspectives on physical activity both during hospitalization and after treatment. These studies highlight the significance of social support by classmates, parents, and professionals, of noticeable physical and psychological effects, and of adapting interventions to the individual situation of each patient. As a final example, within a study on a rehabilitation intervention for preschool children undergoing cancer treatment (including structured active play), Pouplier et al. have examined both the embodied participation of these patients (31), and the perspectives of their parents (32), reporting positive experiences among both.

Notwithstanding the insights gained from the articles cited above, several gaps remain as a function of the limited qualitative insights available. Thus, the purpose of the present study was to examine the perspectives and experiences of childhood cancer patients and their parents, respectively, regarding their participation in a structured and individualized exercise intervention.

## 2 Methods

### 2.1 Research approach

The present study represents a subproject within the multi-center randomized-controlled trial *FORTEe*^1^, which aims to evaluate both physiological and psychosocial effects of a structured and individualized exercise intervention for children and adolescents undergoing cancer treatment. The *FORTEe* trial was registered on ClinicalTrials.gov with the identifier NCT05289739 and in the German Clinical Trials Register with the identifier DRKS00027978. Participants randomized to the exercise group received an 8–10-week exercise intervention supervised by an exercise professional during the intensive cancer treatment phase. The intervention included both in-patient exercise sessions and supervised online sessions (if the patient was at home) and could be supplemented by the use of digital tools. The exercise protocol consisted mainly of age-appropriate, moderate-intensity combined strength and aerobic exercise, as well as flexibility and balance training. Participants in the control group received usual care according to the local standard.

The objective of this subproject was to examine patients' perspectives and lived experiences of participating in the exercise intervention by generating narrative and descriptive data that would complement the standardized data generated in the clinical trial. For that purpose, we conducted qualitative interviews and participant observations with a subset of patients and parents at the Childhood Cancer Center of the University Medical Center of the Johannes Gutenberg University Mainz, Germany (UMC Mainz), one of the ten clinical centers participating in the *FORTEe* trial.

### 2.2 Recruitment and participants

Initially, all patients aged 6–21 years with sufficient command of German language and eligible for participation in the *FORTEe* clinical trial at UMC Mainz were informed about the subproject by the exercise team. After that, the qualitative researcher (D. W.) introduced himself and resolved any questions. With these separate steps, we aimed to reduce the amount of study information given at one time, as childhood cancer patients have to process a great deal of information alongside burdensome treatment experiences and disruptions of their lives (33). Families received printed information and assent/consent forms in age-group specific designs; both patients and parents needed to give written assent/consent. After assenting/consenting, patients were randomized into the intervention group or the control group. As the qualitative subproject focuses specifically on the experiences and perspectives regarding the exercise intervention, only patients from the intervention group were eligible. Patients could also choose to participate only in the intervention without participating in the subproject, and parents could decide independently whether or not they wanted to participate.

### 2.3 Data generation

We employed two qualitative methods of data generation. First, we conducted *participant observations* during exercise sessions (34, 35). Prior to each observation, we asked the patients whether they agreed with the qualitative researcher joining and observing the session. Usually, the exercise team and the researcher met on the ward, picked up the patient from their room and went to the exercise room together. When it seemed appropriate and feasible, the researcher assumed an active role by participating in exercises or competitive games, and this was sometimes even asked for by the patients. No notes were taken during the observations, as this would have conflicted with this active role, as this may have evoked the false impression of “evaluating” the patients' performance. We also avoided recording the observations, as this would not have been practical during the sessions and may have felt intrusive for some participants. Thus, observations were written immediately afterwards. These observations comprised a detailed description not only of the exercises done, but also of social interaction between participants, exercise therapists and the researcher, of participants' statements referring to their current experience, and of situations that appeared ethically significant.

Second, we conducted *qualitative interviews* with patients and parents (36). Following recommendations for qualitative research in pediatric oncology (33), we aimed to give participants as much control as possible regarding the interview setting (time, place, duration, presence of parents), although this was limited given the institutional restrictions and the health status of the patients (37, 38). An interview guide was developed, but used flexibly in view of the individual situation. Interviews with parents also focused on their child's experiences and on the perceived effects of the exercise intervention. To be able to contextualize the interview data, field notes were written after each interview, capturing aspects of the meeting that could not be audiotaped (e.g., reflections on research practice and relationships; atmospheres and moods; events before and after the interview). Interviews were audiotaped (except one interview conducted in the waiting area of the ward, which was not recorded due to privacy reasons) and transcribed in detail.

### 2.4 Data analysis

We chose *reflexive thematic analysis* (RTA) as our method of analysis. RTA, developed by Braun & Clarke (39, 40) and colleagues (41), is a particular version of *thematic analysis* (TA), a qualitative approach “[…] for identifying patterns (“themes’) in a dataset, and for describing and interpreting the meaning and importance of those.” (42). These authors have promoted RTA as a suitable approach to data analysis in research on exercise (42, 40) and on health (43). RTA's flexibility and its orientation towards deep interpretive engagement with the data appeared especially suitable in view of our small and heterogeneous sample. We have used the RTA Reporting Guidelines (RTARG) published by Braun and Clarke (44) when preparing and reviewing this manuscript.

RTA's flexibility requires researchers to actively make and transparently communicate methodological decisions, adopting a stance of “reflexive openness” (44). One key decision involves the epistemological framework. We adopted an experiential orientation, treating participants' accounts as *reflective* of their lived experiences, rather than analyzing them through the lens of social construction. Another methodological decision concerns the layer of meaning on which the analysis takes place. We analyzed both *semantic* (more explicit, “surface”) and *latent* (more implicit, “hidden”) meaning; sometimes, experiences were discussed quite explicitly, whereas other parts of the data required deeper interpretation. Finally, our analysis was primarily *inductive,* i.e., grounded in the data, rather than *deductive* (i.e., starting with predefined codes).

We used the recursive six-step process developed by Braun, Clarke, and colleagues (42). *Familiarization* consisted of detailed interview transcription, reviewing transcripts in anticipation of further interviews, followed by data reading, coding and theme generation. For *coding*, we used the analysis software MAXQDA (2020). We started coding the first data items while data generation was ongoing. We wrote analytical notes for data segments which appeared pertinent, or which required a deeper analysis. Multiple (three) rounds of coding proved valuable. Initially, our codes were too abstract and resembled themes rather than remaining close to the data. To maintain an inductive approach, we refined our coding in the subsequent rounds, ensuring that the codes were more data-driven before moving toward theme development.

Braun and Clarke note that *themes* (“patterns of shared meaning underpinned by a central organizing concept” (40), should be distinguished from *domain summaries* (“summaries of the range of meaning in the data related to a particular topic or ‘domain’ of discussion.”, (40). Drawing on this distinction, we purposefully created four domain summaries in MAXQDA as a first tool to group our codes: *Positive experiences and effects of exercise therapy*; *Limitations of exercise therapy*; *Perspectives and needs regarding the implementation of exercise therapy*; *Impact of illness and treatment*. This was a helpful intermediate step towards *development of themes*, which started systematically as coding was mostly finished. Here, we used a mind-mapping tool, which fostered the creative and flexible visualization of interrelations between codes and themes. We inserted and clustered our codes (as contained in the four domain summaries) and developed themes from them. We maintained recursiveness by returning to the data whenever needed, and by rearranging, rephrasing, merging or discarding codes as well as themes. Once we had generated a set of candidate themes, we proceeded to the fourth phase, *reviewing potential themes,* although these phases cannot be separated strictly from each other. Rather, developing and reviewing themes was a recursive process. *Defining and naming themes* began after we had finalized our themes, although this phase, too, is grounded in the previous two. Refining themes continued even in the final step, *writing* this report. Two peculiarities of our approach to RTA were that we related a few codes to more than one theme, and that we used multiple *central organizing concepts* to grasp the shared meaning of the codes within one theme.

### 2.5 Reflexivity

Reflexivity is integral to our method of analysis and is increasingly regarded as a core value of qualitative research in general (45, 46). We aimed to practice reflexivity from the beginning by writing reflective notes (47) focusing on relationships to the participants and other ethical aspects of the research, as part of the case-related field notes.

Reflecting on positionality, one aspect of our study is the *generational asymmetry* between our adult researcher and our minor patients. This asymmetry entails a power imbalance, as adult researchers usually control most parts of a study (48). However, acknowledging this should not lead to attributing a “victim role” of powerlessness to patients, who may exert their own agency when participating in research (49, 50). A second aspect of positionality is that our researcher was not one of the exercise professionals who saw the patients on a regular basis. This may have influenced the relationship with the patients, and, subsequently, their answers in the interviews. However, we gained the impression that participating in the exercise sessions was an appropriate measure for building trust and for gaining additional insights into patients' experiences.

While we perceive it as important to introduce minors' perspectives into research on pediatric exercise oncology, we refrain from presenting these as allegedly “authentic voices”, in line with our orientation towards critical childhood studies (51) and RTA (39). Thus, the perspectives presented here should be understood as relational products of situated interactions, and within the boundaries of their interpretation by adult researchers, as participants have not been involved in data analysis.

## 3 Results

Between April 2022 and December 2023, six patients (3 male, 3 female) and five parents (3 mothers, 2 fathers) were enrolled (Table 1 shows participants' characteristics). Four more patients would have been eligible, but did not participate because of non-compliance, refusal or withdrawal of consent. Data generation started in June 2022 and ended in February 2024. We conducted 11 interviews (ranging from 7 to 50 min), as well as 10 participant observations, ranging from 45 to 60 min.

**Table 1:** Characteristics of study participants.

Patient characteristics	*n* (%)	Mean ± standard deviation	Range
Age at enrolment (years)		11,67 ± 4,19	6–16
Sex			
Female	3 (50)		
Male	3 (50)		
Diagnosis			
Leukemia (ALL)	4 (66,67)		
Hodgkin lymphoma	1 (16,67)		
Osteosarcoma	1 (16,67)		
└ Thereof relapses	1 (16,67)		
Parent characteristics			
Mother	3 (60)		
Father	2 (40)		

The interviews frequently developed beyond the limits of the thematic focus into conversations about other illness and treatment experiences or life beyond illness, and such conversations also happened during the observations. This seemed important to most participants, whereas some of their answers regarding the experience of the intervention were quite succinct. Thus, there was a great variety in the thoroughness of answers. The information generated from participant observations did not differ from the interviews but rather reinforced and contextualized the findings.

**Figure 1:**
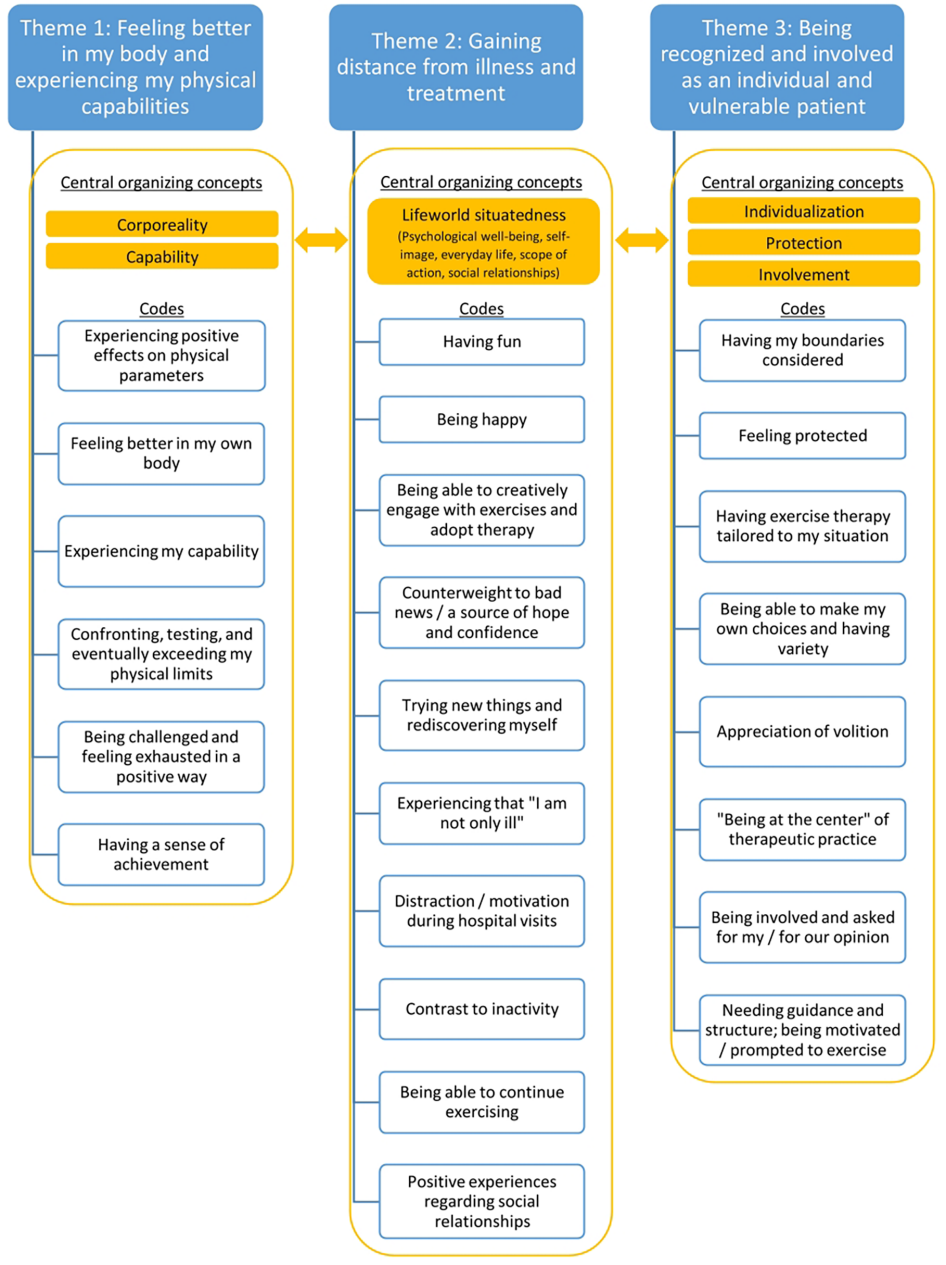
Simplified map of themes, codes and central organizing concepts.

We generated three fully developed themes: 1. *Feeling better in my body and experiencing my physical capabilities;* 2. *Gaining distance from illness and treatment;* 3. *Being recognized and involved as an individual and vulnerable patient*. Figure 1 shows a simplified map of the three themes and their codes and central organizing concepts. We provide a Supplementary Table in which further data extracts are listed together with their assigned codes, from which the respective themes have been generated.

We have deliberately not constructed *themes* from the two domain summaries *Impact of and impairments due to illness and treatment* and *Limitations of exercise therapy*. This is because we believe both the burdens and limitations mentioned by the participants or witnessed by us can hardly be united in relation to a *shared meaning* [Braun & Clarke explicitly mention “Drawbacks of […]” as a typical domain summary which is *not* a theme; (40), p. 593]. However, we present the insights from these domains as two additional result sections: *Background: Impact of and impairments due to illness and treatment* and *Limitations of exercise therapy*. All data excerpts in the following paragraphs have been translated from German specifically for this publication by D. W. and were independently reviewed by M. N. and F. A. (however, the analysis was still conducted in German).

### 3.1 Background: impact of and impairments due to illness and treatment

Throughout our data collection, the impact of illness and treatment on the patients' physical capacities remained palpable. *Physical* symptoms such as pain or fatigue forced patients to reduce or cease their sports activities soon before diagnosis. Consequently, this resulted in impairments on *social* relations:

“[…] I went to the gym three or four times a week with my friends […] or with my brother, that was our thing, our time together during the week and it sucked to drop that completely.”

(16-year-old female patient)

Especially in the first weeks of treatment, chemotherapy exacerbated or caused further impairments of mobility, strength, and endurance. Together with lifeworld constraints such as repeated hospital stays and absence from physical education, this forced patients to cease their sports activities completely for some time. As a result, patients' fitness further deteriorated – here, the “downward spiral” of physical inactivity identified by Grimshaw et al. (7) became apparent – so that daily activities became arduous or even impossible:

“[…] in the first weeks and months, he was really lying around a lot; he could hardly do anything […] he couldn't walk up the stairs on his own.”

(Father of a 7-year-old male patient)

Consequently, the first exercise sessions were physically challenging for most patients:

“[…] I noticed that I hadn’t exercised for quite a long time, […] I didn’t have good endurance and fitness anymore, and that, well, was extremely exhausting.”

(15-year-old female patient)

As patients made progress both with their cancer treatment and with exercise therapy, side effects or illness events (such as fever, pain, mood changes, and impairments related to specific body parts) situationally compromised their capacities to exercise. In some cases *catheters* limited patients' mobility or access to activities such as swimming.

### 3.2 Theme 1: feeling better in my body and experiencing my physical capabilities

Due to the overlap between their codes and organizing concepts, themes 1 and 2 are closely intertwined. The central organizing concepts of theme 1 are *corporeality* and *capability*. Our use of the concept *corporeality* is inspired by phenomenological understanding of the body (52–54). From this perspective, corporeality firstly expresses a dual understanding of the human body as a *material body* that a person *has*; and as the *lived body* that a person *is*. While the former results from an objectifying perspective (e.g., when a person receives medical treatment), the latter refers to the body as part of our existence. Corporeality secondly signifies the fact that our (lived) body is a medium of our relation to the world and our possibilities to act upon it. This perspective serves as a link to the second organizing concept, *capability*, which we integrated into theme 1, as this topic was often addressed in close connection to bodily experiences. Corporeality appears as a suitable concept to grasp the shared meaning of the participants' body-related experiences, in which these very two perspectives find expression as fluctuating yet indivisible aspects.

Participants described various positive effects that exercise therapy has had on a bodily level, primarily on their mobility, strength and endurance (some participants also mentioned increased concentration, motor skills, body control, and the possibility to lose weight as positive effects). These effects contributed to patients' rehabilitation in certain *activities of daily living* again:

“[…] physically, she was reset to zero, and within a few weeks only, she recovered to the point that you can go for normal walks with her; she dashes up and down the stairs on her own again”

(Mother of a 6-year-old female patient)

This mother expresses her surprise about the rather rapid recovery of her daughter's physical fitness. One father was surprised by the stepwise nature of the recovery, realizing that the patient no longer needs to be confined to physical inactivity:

“[…] [there] was this transition period, right? from the time when he needed to lay down on the sofa, to [exercise therapy] becoming a routine, and next it was like: “no, you don’t need to lay down on the sofa anymore, you can [move] again”. and then you noticed how he was able to do quick movements and to be active again during exercising, right? that was hardly conceivable before […]”

(Father of a 7-year-old male patient)

Apart from mentioning positive effects on their child's capacities to carry out ordinary activities, participants emphasized their progress *within exercise therapy* as a positive experience in its own right:

“[…] we always make sure to increase my exercises, and this sense of achievement is really cool, if you notice that you can do more than the week before […]”

(16-year-old female patient)

“[…] I was always looking forward to [exercise therapy], because in the beginning, we noticed that I don’t have a lot of endurance, but it kept getting better, I was able to do the exercises for longer and longer […]”

(15-year-old female patient)

These two patients also emphasized the broader positive effects of exercise therapy on their physical well-being, noting that it made them “feel good” or “feel better” and enhanced their physical awareness. While improvements in mobility, strength, and endurance are both experienced and objectively measurable, the focus here shifts to the *lived body* perspective—where “feeling good” and heightened bodily awareness reflect the subjective dimension of *lived experience*.

The improvement of one's fitness goes hand in hand with positive experiences of confronting, testing, and exceeding the perceived limits of one's physical capabilities. These aspects were often linked to each other in the participants' accounts. *Confronting one's limits* may refer to one's inclination regarding certain exercise, as was the case for this participant:

“Sometimes there are also things which are exhausting and which I don’t feel like doing but which I do anyway.”

(6-year-old female patient)

But even more so, it refers to testing one's perceived physical limits, which seemed important to virtually all patients. For example, one mother described the beginning of exercise as a turning point, noting that her physically restricted daughter surprised her by going beyond the expected physical capacities. She interpreted this as a key moment:

“[…] and I think that was the crucial point for her, right? ‘I need to go beyond what I can actually do, and then I can accomplish a lot’ […]”

(Mother of a 6-year-old female patient)

This aspect often became apparent during participant observations, too:

When he was doing the strength exercise, [the exercise therapist] told him that he should stand up a little straighter in case it got too challenging for him. He answered that he would rather increase the difficulty of this exercise [causing (the exercise therapist) and me to laugh].

(Participant observation with a 16-year-old male patient)

Experiencing their extant or improving physical capabilities through exercise often yielded a sense of achievement for the patients:

“[…] if you realize how much you can actually still do, or how much you can do again, that’s quite cool; like [lifting] weights or cycling for some time or stuff like that […]”

(16-year-old female patient)

The fact that patients wanted to “exhaust” their extant or improving physical capacities and the resulting positive experiences did not only become apparent in view of their impaired physical fitness, but also in view of more specific impairments. One patient was affected by considerable vision impairment as a result of cancer treatment. While they needed some assistance in spatial orientation, they still carried out all exercises and handled the situation competently. However, testing one's limits did not automatically result in exceeding them, but also in understanding them precisely *as limits*:

“[…] but I think that [treating exercise therapy as a challenge] is great because [he/she] always has a motivation for that day, so that [he/she] can say ‘one thing I managed, another thing I didn’t’ […]”

(Mother; patients' age and sex anonymized to prevent identifiability)

### 3.3 Theme 2: gaining distance from illness and treatment

While theme 1 and theme 2 are closely connected, bodily experiences and the concept of corporeality play a less prominent role in theme 2. What comes to the fore, are experiences related to the patients' situatedness within their *lifeworld*, understood, from a social phenomenological perspective (55), as the intersubjectively (as well as spatially and temporally) structured world which is the foundation for one's daily life and lived experiences. Illness, treatment, and the pediatric oncology ward or the hospital as institutions characterized by specific social practices and spatial and temporal arrangements become formative factors for this situatedness. More specifically they impact patients' *psychological well-being* and *self-image*, their *everyday life* and *scope of action*, and their *social relationships.* While *corporeality* is fundamental to this situatedness, it is set aside here as it serves as an organizing concept in theme 1. Various experiences of how exercise therapy can mitigate impairments and negative effects resulting from this became apparent in the participants' reports and in our observations. These accounts conveyed how the program attenuated the formative influence that cancer may have on their everyday lives, and its omnipresence within them, and how it thus served as a space in which patients could *gain distance from illness and treatment*.

It became apparent that exercise therapy positively affected their psychological well-being. Besides nonspecific statements about “feeling good/feeling better”, two different sensations or affective states can be differentiated in the data in this regard: *having fun* and *feeling happy*. *Having fun* is a sensation experienced *during* therapy sessions. As a positive affective state, it has a value of its own for these young patients and may be an important factor for their motivation to participate. Having fun contributed to the participants' anticipation of the next session and made the actual (medical) reason for the next hospital visit fade into the background to some degree.

“[…] she’s having fun and she always craves for the next time, right? so she’s actively looking forward to it; of course, we need to go to the hospital because of – [illness and treatment] but she’s actively looking forward to it, she plans her outfit to make sure she’s wearing sportswear […]”

(Mother of a 6-year-old female patient)

*Play* is a key element of children's lives and development that was implemented by combining the exercises with games, doing competitions, enabling patients to engage creatively with the exercises, and handing out small rewards. This element contributed to having fun:

After they had completed all exercises in the bingo game, [the patient and his brother] were allowed to shoot some hoops, which appeared to be a lot of fun for them. After that, [the patient] was given two stickers (one for this exercise session, one for yesterday's online session), which he and his brother put in his sticker album with great joy.

(Participant observation with a 7-year-old male patient)

*Feeling* or *being happy*, in contrast, appeared as a longer lasting affective state that ensues after the exercise sessions. This becomes apparent in one patient's statement: each time she comes back from exercise therapy, her mother welcomes her “as a happy child.” In the accounts given independently by two parents (of different children), who explained that their children were “all smiles” when returning from exercise therapy, as well as in the following statement:

“I’m always happier after exercising. Each time I return from exercising, I’m happy.”

(15-year-old female patient)

With regard to psychological well-being, two patients also explained how the program served as a counterweight to bad news and as a source of hope and confidence. While one patient compared the positive mental effects of exercise therapy with those of social support from her school class and experienced both these aspects as beneficial for her recovery, in the accounts given by another patient this felt benefit was often closely related to experiences of extant or increased physical capabilities (see theme 1):

“[…] if you realize that you can still do a lot of things, that always [motivates you], like ‘[…] you’ll get well again, everything will fall into place, and you can still do so much.’ […] that you’re not only presented with the problems you have at the moment, and with what can go wrong, but that you get [to see] some positive aspects […]”

(16-year-old female patient)

The positive experiences made in exercise therapy potentially affected the patients' *self-image*. As one patient expresses poignantly, exercising helped her realizing that “[…] I am not only ill […]” and that “[…] I am not only this illness.” (16-year-old female patient) These statements point to the fundamental, even existential effect that cancer can have on patients' identities – “being one's illness” – and to the potential of exercise therapy to mitigate this effect. Beyond that, the accounts of one young patient and her mother, who felt that her daughter “discovers herself in a new way” and “develops through the exercise program”, even implied experiences of *personal development* through testing one's boundaries and trying out new activities:

“Sometimes there are things which I don’t feel like doing, but then I do them anyway, and then I realize that I like them. […]”

(6-year-old female patient)

This further reflects how exercise therapy served as a space of restored or expanded *scope of action* for these patients. This scope tends to be impaired due to the restrictions imposed by health issues, treatment regimens and by the ward or the hospital as social institutions. What contributed to this scope was the fact that some patients were able to adopt the exercises creatively and to realize their own ideas (see also theme 3). Sometimes this even appeared to stimulate their (especially younger children's) imagination:

[She] seemed to be in a very good mood, and as it appeared to me, the fact that she 'stepped out of line' during the exercises a few times, using the equipment in a different manner or moving about the room freely, was indicative precisely of this. After this exercise, we removed the course, and [she] hid a small ball […] in the cones and told us that she needed to hide this “gemstone” […]

(Participant observation with a 6-year-old female patient)

This passage also reflects how children sometimes implemented elements of play on their own. Further, participants highlighted how exercise therapy got them “out of their room/bed” and how it served as an activation or counterbalance to idleness, against the background of imposed physical inactivity. This was also a reason for some patients to participate in the first place:

“I just wanted to exercise. I didn’t just want to sit idle and do nothing – although I do that anyway. But I wanted to exercise, that was the main thing.”

(16-year-old male patient)

Moreover, many participants referred to exercise therapy as an opportunity to *continue sports*, i.e., the role that sport used to play before illness. While this may refer to a bodily need or to maintaining one's physical capacities, it may also concern one's *self-image*: if exercising has been part of a patient's everyday life and even of their “identity”, not being able to exercise over a longer period may be a disruptive experience. Exercise therapy may serve as a space that softens this disruption and fulfils a “bridging function”, preventing patients from losing touch with exercising as part of their self-image and lifeworld.

“I think [by participating in FORTEe] I don’t lose joy in exercising. And [I think] that I can resume my own sports activities more quickly, once I’m doing well again, like going to the gym.”

(16-year-old female patient)

“So the main reason [for participating in FORTEe] was that I had exercised before, I was in a gymnastics team at our turnverein […]”

(15-year-old female patient)

Against the backdrop of these experiences, exercise therapy became, as one mother (of a 10-year-old male patient) expresses it repeatedly, “an important building block” within the patients' everyday lives with illness and treatment, to the extent that they were actively anticipating and planning their next exercise sessions:

“[…] I had something to look forward to during the day, especially during chemo when I wasn’t allowed to leave the ward anyway, and as soon as the chemo was finished, one of the [exercise therapists] dropped by, and I was always looking forward to this […]”

(15-year-old female patient)

There were no signs of patients experiencing the sessions as an obligation. One patient appreciated online therapy sessions (performed at home) as a positive “task” that gave structure to her everyday life. Most participants described exercise therapy as a *distraction* from, or as a *counterbalance* to their strenuous treatment routine and life on the ward, which lost their severity to some extent. Consequently, it actually became easier for the families to come to the hospital.

“[…] when we’re having our appointments in the hospital, and we know that we can go to exercise therapy afterwards, that’s a good motivation for him to start the day in a good mood […]. then he’s already planning, ‘oh, but then I could go see [the exercise therapists] afterwards’, and anything that needs to be done before isn’t such a big hurdle anymore.”

(Mother of a 7-year-old male patient)

Exercise therapy also became a welcome element of everyday life with illness and treatment due to its implementation as a *social* or *shared activity*. First, this social dimension appeared to be appreciated as a value of its own by the participants, who seemed to enjoy the presence and participation of the exercise therapists and the possibility to chat with them or to engage in competitions, as well as the option to invite along parents, siblings or other patients to the sessions:

“[…] I think it’s cool that [the exercise therapists] participate in the exercises, that we get in kind of a ‘battle’, ‘how much can you do, how much can I do?’ or that they’re like ‘we do it as a team now so that you’re not being watched all the time on your own […]’.”

(16-year-old female patient)

Second, this implementation of exercise therapy as a social or shared activity enabled more specific positive experiences, such as being encouraged during the exercises, receiving recognition or praise for one's performance, or experiencing the pride of one's parents. These experiences may in turn have had positive effects on the patients' self-image:

[…] [Putting stickers into his FORTEe booklet] seemed to be fun for him, and he eventually said that he would show the booklet to his parents and siblings, adding that “then they are proud of me.’

(Participant observation with a 10-year-old male patient)

[…] [He] was giving high fives with his dad after he had completed an exercise […] [and] clearly showed his joy when [the exercise therapist] praised him for [completing] an exercise […]

(Participant observation with a 7-year-old male patient)

On the other hand, some families also reported benefits from decidedly using the exercise sessions as a time-and-space of *separation* from each other. During illness and treatment, parent-child relationships are shaped by increased dependence of children on their parents and by additional time spent together. Time spent apart during the exercise sessions potentially *relieved* these relationships. Some participants considered time spent apart important for facilitating children's *independence* from their parents.

“[…] I accompany him everywhere, we’re together twenty-four-seven, so he needs this independence. […] he goes there on his own and that’s okay and important […] for me as a relief to relax, and for him it means independence.”

(Mother of a 10-year-old male patient)

For one young patient (who only allowed her parents to accompany her to a session if her blood levels were not that good), this independence was not merely an abstract concept that parents insist on, but a concrete and important subjective experience:

“No, [mum] shouldn’t come here anymore because I can do that on my own.”

(6-year-old female patient)

### 3.4 Theme 3: being recognized and involved as an individual and vulnerable patient

We constructed this theme from codes that we had first clustered within the domain summary “*Needs and perspectives regarding the implementation of exercise therapy*”. This domain summary concerned questions about how the program should be put into practice, and what needs participants have in this regard. However, we noticed that important information in view of our research interest – the *experience* of participation – is conveyed in these codes, which is why we grouped them in relation to their shared meaning. As a result, we generated three closely related organizing concepts: *individualization, protection,* and *involvement*. Thus, we “promoted” the (*evaluative*) domain summary to a (more *experiential*) theme: *being recognized and involved as an individual and vulnerable patient*.

The three organizing concepts of this theme cannot be disentangled but overlap and refer to each other. However, *individualization* may be regarded as its “superordinate” organizing concept. It refers to the experience of receiving exercise therapy that is responsive to one's individual situation and needs, and of being at the center of therapeutic practice as a patient in a vulnerable life situation. This, in turn, involves *protection against* (or *freedom from*) potential negative experiences or effects, and having exercise therapy conducted, to some extent, in an open, flexible manner that facilitates positive experiences through co-creation, choice, creative adoption and a resulting variety. We have summarized these aspects under the term *involvement*.

Regarding the first aspect, both patients and parents positively emphasized the feeling of being protected against harm during the exercise sessions (as well as when they/their child experienced discomfort during a session), having their boundaries respected in case they/their child did not want to or were unable to participate in a session or perform a specific exercise, and the general volition of the program:

“[…] so I know that when your colleagues exercise with her, they take care of her, [and] the [exercises] that are being done, they fit the physical state […] so, she’s not overchallenged but just exhausted, right?”

(Mother of a 6-year-old female patient)

“[…] due to the port, I wasn’t able to fully raise my arm for some time, so we dropped the [exercises] on that side or did something else that didn’t put as much pressure on the port.”

(15-year-old female patient)

“[…] they don’t force anyone, so it’s really – you notice that it’s voluntary, that it’s an offer, and that it’s meant to support you.”

(16-year-old female patient)

As can be seen from these quotes, the patients' physical vulnerability resulting from illness and treatment often formed a (sometimes explicit, sometimes implicit) point of reference when participants talked about positive experiences of feeling protected or having their boundaries considered.

Regarding *involvement*, the participants valued the possibility to co-create the exercise sessions to some extent, the freedom to sometimes choose their exercises according to their individual preferences, mood or physical capacities, and the experienced variety resulting from that. Co-creation was not restricted to choosing or helping to prepare the exercises, but also to the general setting of the sessions, as some patients would turn on their own music or bring their parents or siblings to the sessions when they were visiting. In this way, they integrated familiar elements of their lifeworld into exercise therapy. Involvement was actively encouraged by the exercise therapists but also happened through spontaneous and creative initiative by the patients themselves. This personal initiative and the concomitant experiences became much more tangible through participant observation than through explicit thematization in interviews:

After some time, [he] […] suggested that he could shoot some hoops while cycling, whereupon I positioned the basketball hoop. Throwing the ball seemed to visibly distract him from the physical effort, which involved pain in his legs, and he continued until he had scored fifteen times.

(Participant observation with a 10-year-old male patient)

Two parents even mentioned the accompanying research (which included questionnaires and half-structured interviews in addition to the open qualitative interviews) as a valuable feature of the clinical trial. In this regard, one mother described it as a positive experience that her daughter's perspectives are actively asked for, but also referred to the need to advance scientific knowledge:

“[…] it’s fun for her that her own opinion is asked for, so that it’s not mum or dad saying ‘exercising is good’ or ‘go sit on your bike, that’s great’ or something, but that she can say ‘how do I feel when I do that?’, ‘am I having fun or am I not having fun?’, so that she herself is getting asked. […] and the interviews are part of it because that includes research as a topic, right? so that [this topic] can move forward […]”

(Mother of a 6-year-old female patient)

Taken together, it appears that these experiences of protection and involvement have contributed to the positive feeling of *being at the center* of therapeutic practice:

“[…] I really like that all the people [i.e., the exercise team] really focus upon me […]”

(16-year-old female patient)

This feeling was sometimes expressed in view of timing, in that participants welcomed the fact that exercise therapy was provided regularly and flexibly. However, it was also expressed in view of gratitude for the time that therapists took with them. Putting the individual patient at the center of therapeutic practice was also implied as an important element by two mothers who talked about their children going to exercise therapy without them. They deemed this situational separation necessary so that exercise therapy could really focus on their children:

“[…] and she unbends because then, it’s about her and not about her parents, right? it’s about her as a person […]”

(Mother of a 6-year-old female patient)

“[…] and that’s important for you [i.e., the exercise team] in that moment, so that you can calmly work with him alone […]”

(Mother of a 10-year-old male patient)

While individualization and its more specific aspects such as unrestraint and involvement appeared to be experienced positively, some patients also voiced appreciation of *being prompted* to exercise and of *receiving clear input* and *targets* from the therapists:

“[…] I think if [the exercise therapists] wouldn’t come up to me, I would exercise less and would rather let myself go and fall into a lazy mode, that’s why I appreciate their commitment and the impulse behind it.”

(16-year-old female patient)

“[…] my concern with exercising is that I have a coach, and they tell me how to do it.”

(10-year-old male patient)

“[…] sometimes I would do what I like, but sometimes one should simply do what [the exercise therapists] say.”

(16-year-old male patient)

### 3.5 Limitations of exercise therapy

We have divided the identified limitations into three different categories: (1.) Factors limiting participation in exercise therapy; (2.) Discomfort caused by exercise therapy; (3.) Limitations of positive effects of exercise therapy.

(1) Most obviously, the bodily and emotional impacts of illness and treatment limit patients' physical as well as motivational capacities to participate in exercise therapy, and this has become apparent in all six cases. Pain, “not being in the mood”, fatigue, and side effects have been the main reasons for patients to be reluctant whether they should participate or not, or to decide to not participate after all:
  “[…] this week I actually told [the therapists] that I don’t feel like exercising, and last week I cancelled it once as well, because I couldn’t do it because I was pretty exhausted.”
  (16-year-old female patient)

In view of such situations, patients valued having their boundaries respected (see theme 3), but sometimes, when they agreed to participate in a session despite previous reluctance (after therapists and/or parents had tried to motivate them), they were also happy about it afterwards. While not explicitly talking about it in terms of “fear”, the youngest participant also recounted that she did not want to exercise in the beginning of her participation and that she cried because of that, but that she lost this aversion after the first few sessions.

(2) There were very few situations in which the patients' vulnerable health condition interfered with exercising, causing temporary physical discomfort or stress (abdominal pain; headache or vertigo; nausea; one fall resulting in a minor bruise) during and after the exercise sessions. Referring to an online training session, one patient recounts:
  “[…] that day, I wasn’t feeling so well and we needed to stop [the session] because I started to feel queasy […]”
  (15-year-old female patient)

Depending on the severity of these situations, the exercise therapists informed the physicians who either looked after the patient directly or ordered further examinations after the session. In view of this, participants reported feeling that they were in good hands. The patients themselves also occasionally referred to their vulnerability during the exercise sessions by explaining that they cannot carry out a specific exercise or that they need to go slow or be careful when doing so. The reciprocal relation between *experienced discomfort* and *barriers to (further) participation* also became visible when one patient reported that, following a fever and an infection, she reduced exercising within the program because she had recently felt very exhausted by it.

(3) There were only two instances in which participants explicitly reported limitations of positive psychoemotional effects, highlighting how these effects are situationally overshadowed by the burdensome nature of cancer and cancer treatment. As one patient expressed it,
  “[…] on days when everything is crap, even [exercise therapy] doesn’t help.”
  (15-year-old female patient)

Similarly, looking back at an especially burdensome phase of treatment, a father explained that the positive impact the exercise therapy had on his son's mood would be of limited duration, until awareness of that burdensome phase prevailed. Beyond these limitations, when being asked explicitly, most participants had no criticisms or suggestions for improvement of the program, apart from ideas to incorporate certain types of sport and from spatial aspects such as having a larger exercise room or exercising outside.

## 4 Discussion

### 4.1 Discussion of results

This study expands the emerging knowledge of childhood cancer patients' experiences of exercise therapy during treatment. Participants reported, and we witnessed mostly beneficial effects on well-being and life situations, as well as positive experiences with the implementation of the program, in contrast to the minor limitations we observed in terms of barriers to participation, experienced discomfort during exercise sessions and limited psychoemotional benefits.

Patients experienced positive effects on the level of *corporeality*, i.e., on their physical capacities such as strength, endurance and mobility, as well as on their physical awareness and well-being (theme 1). Exercise therapy served as a space in which they were able to train and experience these capacities – an experience that is usually severely restricted by cancer treatment and the institutional environment (7, 18, 29). Confronting and potentially exceeding the perceived limits of one's physical capacities appeared to be a positive experience for patients. Thus, exercising contributed to a gradual improvement of the ability to carry out activities of daily living again and yielded a sense of achievement. These results are relevant in view of the impact that impaired physical functioning can have on childhood cancer patients' quality of life and self-esteem (56).

Exercise therapy further became an important element of the patients' *lifeworld* and their situatedness within it, and enabled them to step back from ever-present illness and treatment (theme 2). It allowed for positive sensations such as having fun – not least through elements of play – and feeling happy, became a source of confidence and personal development, and even enabled one patient to revise her self-image as a person who *has* cancer, but who defies being reduced to their illness. This is relevant in view of the significant impact that a cancer diagnosis can have on a child's or adolescent's identity and its formation (57). The program provided patients with a scope of action and served as a counterweight to inactivity, as an opportunity to maintain their relations to exercising, and as a distracting and motivating element of their hospital visits. Moreover, participants appreciated exercise therapy as a shared activity, which appears valuable given the fact that illness and treatment may impair social functioning and relationships of some childhood cancer patients (58, 59). Some participants valued the time apart during exercise therapy as a relief in their parent-child relationships and an opportunity to foster children's independence. Finally, participants appreciated the program in view of *individualization, protection* and *involvement* as “pillars” of its implementation (theme 3). They welcomed participating in a program that was tailored and open to their individual needs and interests, feeling safe in view of their vulnerability, having their boundaries considered, and being able to co-create the sessions and contribute their perspectives.

There were a few minor limitations to these positive results. First, illness events and fluctuating health status and mood sometimes impeded participation in exercise therapy, and one young patient reported initial reluctance to participation. These factors reflect aspects of acceptability, as patients' willingness and motivation to engage in exercise therapy might be influenced by their physical and emotional status. Second, the patients' vulnerable health status occasionally interfered with exercising. Leading to rare instances of physical discomfort. One patient reported reducing participation after experiencing exhaustion due to a prior fever and infection. These challenges relate to feasibility, as they highlight practical constraints in maintaining consistent participation. Third, the burdensome nature of illness and treatment occasionally overshadowed the experienced psychoemotional benefits.

These results are in line with existing qualitative research on physical activity interventions in childhood cancer patients, which has also reported largely positive experiences (18, 7, 30–32). Some specific aspects have also been identified by these studies, e.g., the sense of achievement through challenging one's limits (31); the significance of having fun (18, 31, 32); the role of activity interventions as a motivating counterweight in everyday hospital life (18, 31); the appreciation of such interventions as social activities (29, 30); and positive effects on parent-child-relationships (31, 32). Positive experiences have also been shown in qualitative studies with adult cancer patients (60–62), although different populations should be distinguished from one another according to their specific burdens and needs.

### 4.2 Strengths and limitations

Our study has specific methodological strengths. The combination of qualitative interviews (with both patients and parents) and participant observations generated rich and insightful data. Keeping the interviews *open* enabled participants to report on aspects important to them and build rapport with the researcher. Participant observation turned out to be a fruitful method of data generation, as pediatric exercise oncology is a setting of *embodied and performative practice*, and some important phenomena and experiences related to it may elude retrospective verbalization in interviews. Further, participant observations enabled us to gain insights into the patients' *situated* participation experience (and practices) which may have otherwise been obscured. At the same time, the researcher's active role in some exercises may have influenced interactions with patients. While this approach helped build a relationship and encouraged engagement, it may also have introduced an observer effect, which should be considered when interpreting the findings. Regarding the analysis, using RTA produced a comprehensive and nuanced interpretation of the data.

There are also limitations of this work. The small sample of participants who all received care in the same German hospital means that results might not be generalized to the wider childhood cancer population. Beyond the single-center setting, sociocultural differences—such as attitudes toward physical activity during illness, the perceived role of rehabilitation in pediatric oncology and the integration of structured exercise programs into supportive care—may further limit the transferability of our findings. Additionally, differences in healthcare infrastructure, available resources, access to specialized exercise professionals and family expectations regarding medical and supportive care may influence how such interventions are received and implemented in other settings. Moreover, the patients' answers varied greatly in their detailedness (here, it should also be noted that one interview couldn't be recorded because it took place in a waiting area, so that a protocol had to be written from memory). Consequently, the perspectives of individual patients are represented unequally in our study. There are also sources of bias in our data. First, participants may have been inclined to emphasize positive experiences or give socially desirable answers because our researcher, albeit not an exercise scientist, was a member of the study team, which possibly made it more difficult to talk openly about limitations. Second, the predominantly positive responses may partly be a result of a selection bias, as the families who agreed to participate in the study may be those who have already had an affinity for exercising, and as all patients reported that they had done sports before their illness. Thus, the potential of the program to positively influence a patient's basic attitude towards physical activity needs to be further investigated. Third, our position as professionals working in the field of pediatric exercise oncology may have influenced interpretation and coding of the data, and thus the presented results. However, we attempted to maintain a critical distance by using a strongly reflective method of data analysis and by highlighting possible limitations of this subproject above.

### 4.3 Implications for practice and research

The results of our study suggest that an expansion of exercise therapy as part of their standard care could be beneficial for many childhood cancer patients. We are aware, however, that this is a matter of resources – both in terms of funding and material and spatial prerequisites, as well as of culture, as such an expansion requires a general positive attitude towards physical activity within institutions (7). Incorporating exercise therapy as a crucial element of supportive care in policies such as the World Health Organization's *CureAll* framework (63) could foster change to that effect. Efforts are also being made at a national level, in Germany, to provide quality-assured, i.e., guideline-based (64), exercise therapy in all pediatric oncology centers and to raise awareness among treatment teams, specialists and families for the general promotion of physical activity (64).

Regarding its implementation, our findings indicate that exercise therapy is best put into practice as a flexible offer that is sensitive to the needs and vulnerabilities of individual patients and that leaves room for them to creatively make the sessions “their own”, while at the same time providing structure and consistency so that patients can reliably benefit from it in the long run. This is in line with existing results and recommendations (7, 18, 23, 24). As indicated above, *individualization, protection* and *involvement* may serve as helpful guiding principles informing the implementation of future interventions. They are also relevant in view of the minor limitations we noted. Involved professionals should identify barriers to participation and work with families to overcome them where possible. However, while motivating patients is important, their boundaries must always be respected, and reluctant patients should be given time to get used to interventions. Furthermore, exercise therapists should be sensitive to potential experiences of discomfort and stay in close contact with medical team members.

In future research in the field of pediatric exercise oncology, qualitative methods should be incorporated as an integral part, as they allow for in-depth explorations of the *lived experiences* of those affected. This is crucial because exercise therapy is not only a matter of physiological parameters (24). Future qualitative studies could broaden the knowledge by involving further agents (e.g., other family members; health care professionals) as participants and by integrating additional (“creative”) methods of data generation that are responsive to the needs of different patients, as well as participatory approaches that involve families in the design of studies and interpretation of results (20).

## 5 Conclusion

This qualitative study revealed predominantly positive experiences of childhood cancer patients (and their parents) regarding their participation in a structured and individualized exercise intervention. Participants reported and we observed significant positive effects on their physical and psychological well-being, on their physical capabilities and capacity to act, and on their everyday life with illness and treatment. These positive effects and experiences highlight the appropriateness of expanding exercise therapy or other physical activity interventions as crucial elements of supportive care of childhood cancer patients. Such interventions should be implemented as flexible, socially responsive activities that are tailored to each patient's individual situation and needs.

## Data Availability

The datasets presented in this article are not readily available because qualitative research data contain personal information of participants and cannot be fully anonymized, and in order to comply with the European and national data protection regulations applicable for this project. Requests to access the datasets should be directed to Dennis Wilke, denwilke@uni-mainz.de.

## Footnotes

^1^
Full title: *Get strong to fight childhood cancer – An exercise intervention for children and adolescents undergoing anti-cancer treatment.*
