# Supplementary material for: Childhood cancer patients' experiences of a structured exercise program. A qualitative study using reflexive thematic analysis
Source: Front Pediatr. 2025 Mar 26;13:1547822. doi: 10.3389/fped.2025.1547822 (PMC11979272; doi:10.3389/fped.2025.1547822)
Supplement: Supplementary file 1 [file Table1.pdf]

SUPPLEMENTARY TABLE – Further data excerpts and their assigned codes

Please note:

- This table contains further data excerpts on the three themes and two domain summaries developed from our analysis, presented in the same order as they appear in the manuscript.
- In reflexive thematic analysis (RTA), a differentiation is being made between fully developed *themes*, understood as "patterns of shared meaning underpinned by a central organising concept" and *domain summaries*, which are "organised around a shared topic but not shared meaning".[1] Thus, we have deliberately not constructed *themes* from the two domain summaries *Impact of and impairments due to illness and treatment* and *Limitations of exercise therapy*, as we believe that both the burdens and limitations mentioned by the participants or witnessed by us can hardly be united in relation to a shared meaning.
- All data excerpts in this table have been translated from German specifically for this publication. The analysis was conducted with the original German data.
- This table does not contain *all* coded segments within our data, but a selection of meaningful passages that is intended to enhance the transparency of our analysis.
- Square brackets contain pseudonymizations, omissions, or words that have been added during the translation to make the statement comprehensible.
- Data excerpts from participant observations (and one interview protocol) are *italicized*.

[1] Braun V, Clarke V. Reflecting on reflexive thematic analysis. *Qualitative Research in Sport, Exercise and Health*. (2019). 11(4):589–97. doi: 10.1080/2159676X.2019.1628806.

3.1 Domain summary 1: Background – Impact of and impairments due to illness and treatment		
**Data excerpt**	**Data source, participant**	**Codes assigned**
Also relatively early on, [she] explained to me that she could neither go swimming (due to the port catheter) nor go on vacation at the moment. [She] explained the impossibility of going on vacation citing the possibility of going to the emergency room, which is incompatible with travelling further away.	Interview (not recorded, protocol written from memory),<br>6-year-old female patient	• Illness and treatment as restrictions of social relations<br>• Negative effects of illness / treatment on one's scope of action / capacity to act<br>• Illness and therapy restrict the ability to be physically active / do sport
"[…] she could no longer walk, climb stairs, go to the restroom on her own […]"	Interview,<br>Mother of a 6-year-old female patient	• Negative effects of illness / treatment on one's scope of action / capacity to act
"[…] because people just associate you with this disease, […]"	Interview,<br>16-year-old female patient	• Negative effects of the illness on one's self-image
"[…] and when people looked down on you like that and […] it may have been a super nice look, but I just didn't like it."<br>[This is about a period of the illness / treatment when the patient was in a wheelchair.]	Interview,<br>16-year-old female patient	• Negative effects of the illness on one's self-image
"I think it was when I couldn't walk? When I had the severe pain; I [...] was dependent on a wheelchair for three weeks, and that really annoyed me, because I'm a person who doesn't like to ask for help, and I was dependent on other people."	Interview,<br>16-year-old female patient	• Negative effects of illness / treatment on one's scope of action / capacity to act<br>• Negative effects of the illness on one's self-image
"At first I was really annoyed that I had to stay here for so long, because I'm sixteen, it's my summer vacation, and all my friends are on vacation and on the move."	Interview,<br>16-year-old female patient	• Illness and treatment as restrictions of social relations

"[…] because before I came here, I hadn't been able to exercise for so long because I had these knee problems."	Interview,<br>15-year-old female patient	• Illness and therapy restrict the ability to be physically active / do sport<br>• Negative impact of illness and therapy on fitness, mobility, strength and endurance
"I would love to go to school but I'm not allowed to. I actually really like going to school because, it sounds weird, but I enjoy school."	Interview,<br>15-year-old female patient	• Illness and treatment as restrictions of social relations
"[…] Of course he's slowed down in a lot of things now, including swimming or things like that […]. Due to the catheter he says 'no, because the tube sticks out of the chest' […]. Then it has to be taped and he doesn't like bandages […]. There is the fear it might get infected and then it becomes about the disease again."	Interview,<br>Mother of a 10-year-old male patient	• Illness and therapy restrict the ability to be physically active / do sport
"Yes. That was also back then [year], he used to play soccer outside with the children behind the door, but not often, because he was afraid someone might rip the central line out"	Interview,<br>Mother of a 10-year-old male patient	• Illness and therapy restrict the ability to be physically active / do sport<br>• Illness and treatment as restrictions of social relations
"Well, in the beginning you couldn't do that much. When you still had the cortisone and your muscles were suddenly so weak, do you remember? We couldn't really get into the room with [exercise therapist 1] and [exercise therapist 2]. We drove over there in the wheelchair, then you did some exercise while sitting, while lying down."	Interview,<br>Mother of a 7-year-old male patient speaking to her son	• Illness and therapy restrict the ability to be physically active / do sport<br>• Negative impact of illness and therapy on fitness, mobility, strength and endurance
"So we definitely noticed a lot of muscle loss, but that was in the initial phase when he was admitted here and immediately after the treatment he was physically weakened by the therapy and we hadn't even started [exercising] with you yet […]."	Interview,<br>father of a 7-year-old male patient	• Negative impact of illness and therapy on fitness, mobility, strength and endurance

3.2 Theme 1: Feeling better in my body and experiencing my physical capability<br>Central organizing concepts: Corporeality; Capability		
**Data excerpt**	**Data source, participant**	**Codes assigned**
"[…] you can see this very clearly in the training sessions, she comes back with a very, very bad hemoglobin value, pale as cheese and exhausted but smiling all over her face."	Interview,<br>Mother of a 6-year-old fe-male patient	• Being challenged and feeling exhausted in a positive way<br>• Being happy
"[…] I like to set myself goals and I also like to achieve things […] when I get bad news or something like that during the illness, and I then realize how much I can actually still do or can do again, that's really pretty cool; like with weights or when I can still ride a bike for so long or things like that, that always gives [me] a glimmer of hope that everything will be okay again."	Interview,<br>16-year-old female patient	• Having a sense of achievement<br>• Counterweight to bad news / a source of hope and confidence<br>• Experiencing my capability<br>• Experiencing positive effects on physical parameters
"I often have sore muscles afterwards, because I realize that my legs just aren't used to much exercise anymore, or my arms; but it makes you feel good when you notice the next day that you've done something. But also, above all, moving in general […] I actually find it positive, yeah."	Interview,<br>16-year-old female patient	• Negative impact of illness and therapy on fitness, mobility, strength and endurance<br>• Being challenged and feeling exhausted in a positive way<br>• Feeling better in my own body
Meanwhile, I noticed that [she] completed this exercise with a noticeable 'determination' […]. She could have finished earlier - given the visible exhaustion - but seemed resolved to complete the exercise. This made me think that exercise therapy can also offer children and young people the opportunity to experience a contrast to their undeniable bodily limita-tions.	Participant observation,<br>15-year-old female patient	• Confronting, testing, and even-tually exceeding my physical limits<br>• Having a sense of achievement<br>• Experiencing my capability<br>• Being challenged and feeling exhausted in a positive way
"[…] and that just, that somehow makes you feel better in your body and, somehow also makes you fitter, […] you feel better afterwards."	Interview,<br>15-year-old female patient	• Feeling better in my own body<br>• Experiencing positive effects on physical parameters

"[…] you build up your muscles when you exercise, and then at some point you have better control over your body; you can do more […] you have more strength or […] more endurance, things like that"	Interview,<br>15-year-old female patient	• Experiencing positive effects on physical parameters<br>• Feeling better in my own body<br>• Experiencing my capability
Again, he was hesitant, mentioned the pain in his leg, and when [the exercise therapist] asked him if he felt confident exercising (or with how much weight), he mostly replied 'I don't know'. [The exercise therapist] then suggested again that he could see how far he could go, and again he did all the exercises without any problems: he even did more repetitions than planned of his own volition on the cable pulley; then he did bench presses with two single dumbbells in his hands, where he seemed very unimpressed by the weight and increased it from set to set; then he did seated bicep curls; and finally he went onto the mat for two more exercises.	Participant observation,<br>10-year-old male patient	• Confronting, testing, and eventually exceeding my physical limits<br>• Having a sense of achievement<br>• Experiencing my capability
"Basketball or rowing or I don't know, he always motivates himself, so he's usually like 'I've mastered this and that' "	Interview,<br>Mother of a 10-year-old male patient	• Having a sense of achievement<br>• Experiencing my capability
"No, it was just that for me the focus was on what was important, which I know from the first therapy, movement did him good, so for the muscles, for everything, for the motor skills, […] I trusted you completely."	Interview,<br>Mother of a 10-year-old male patient	• Experiencing positive effects on physical parameters
"[…] and you could really tell during strengths exercises, [that] he had hardly any strength; so you [the exercise therapists] would always be surprised and would say, 'Wow, now you've got real strength in your legs'; he had more strength to show than they [the exercise therapists] would have expected, right? So, it's a sign that not all the muscles were gone yet. […] he's strong too, isn't he?"	Interview,<br>Father of a 7-year-old male patient	• Confronting, testing, and eventually exceeding my physical limits<br>• Having a sense of achievement<br>• Experiencing my capability

3.3 Theme 2: Gaining distance from illness and treatment<br>Central organizing concept: Lifeworld situatedness		
**Data extract**	**Data source, participant**	**Codes assigned**
The parcours [would have] actually been over at this point, but [she] extended it here independently: at the end of the upturned bench was a stack of round plastic 'mats', each with three-dimensional surfaces with different patterns that could be felt when stepped on (e.g. a surface with many small nubs, similar to a massage ball, which [she] described as her favorite mat and said to [exercise therapist 1] that she should also stand on it). [She] now took these mats and independently built a path to another climbing arch that was in the room but had not been built into the parkour by [exercise therapist 1].	Participant observation, 6-year-old female patient	• Being able to creatively engage with exercises and adopt therapy
"[…] I think that [she] is now rediscovering herself through her exercise program, what she says and how I experience her […]. I can imagine that she gets a lot out of it for herself and has a lot of fun discovering new things […]. Suddenly, handball became a topic […]. I do believe that through the exercise program she also develops a bit and tries out other things"	Interview, Mother of a 6-year-old female patient	• Trying new things and rediscovering myself
"[…] and I can just see the difference in our daughter, she's so exhausted she can't even get to the car anymore, understandably because she's completely exhausted, but she's all smiles"	Interview, Mother of a 6-year-old female patient	• Being happy<br>• Being challenged and feeling exhausted in a positive way
"I also think it's cool when I'm at home and we have online training so that I have an appointment every day. I can then think, ah, you've got something else, like my tennis training back then […] I think that's actually quite cool."	Interview, 16-year-old female patient	• Contrast to inactivity<br>• Being able to continue exercising
"[…] and then we sat down at home and did some strength exercises and a bit of stretching and stuff, so I didn't have time to just laze in bed but I could just do the training at home"	Interview, 16-year-old female patient	• Contrast to inactivity

"It's just that when I do something, when I exercise, I realize that I'm not just ill; because people associate you a lot with this illness, and if you exercise then you show yourself that you're not that ill. And I think that was important to me, or is important to me, that I continue to exercise a lot, that I go out a lot, that I don't hide away, that I'm still present and that people notice that I'm not just this illness."	Interview,<br>16-year-old female patient	• Experiencing that "I am not only ill"<br>• Contrast to inactivity
"and we talked together and listened to music; it was actually quite cool, as if I was in physical exercise class myself"	Interview,<br>16-year-old female patient	• Positive experiences regarding social relationships
"It [also] became apparent how much he enjoyed exercising, as he asked several times for an extension and also enquired with [the exercise therapist] beforehand about how long he could theoretically exercise. In any case, it seemed that he didn't want to stop"	Participant observation,<br>10-year-old male patient	• Having fun
" […] and I'm actually a person who moves quite a lot, so I thought FORTEe was really cool so that I could keep exercising"	Interview,<br>16-year-old female patient	• Being able to continue exercising
When we were ready to leave […] his mother joked that it would do them both good to be separated from each other for an hour and that she could calm down herself during that time.	Participant observation,<br>10-year-old male patient	• Exercise therapy as a relief for parent-child-relationships [This code has been integrated into the code *Positive experiences regarding social relationships*]
"Exactly. He really enjoys it and especially when we're here on the ward and the [exercise therapists] encourage [him] to exercise. He's into it straight away because it distracts him from everyday chemotherapy routine and for him it's a very, very important component"	Interview,<br>mother of a 10-year-old male patient	• Distraction / motivation during hospital visits<br>• Having fun
" […] this lifts him up, throughout therapy; […] the children lie in bed all day anyway and are taken out of it, with exercising"	Interview,<br>mother of a 10-year-old male patient	• Contrast to inactivity
[He] then reported that he either went to the gym or had football training every day. A detailed conversation about football developed between the three of us [...] and it seemed as if this or exercising in general was an important leisure activity for [him].	Participant observation,<br>16-year-old male patient	• Being able to continue exercising

"Yes, well, I can communicate well with them, they are also really nice and [considerate], yes."	Interview,<br>16-year-old male patient	• Positive experiences regarding social relationships<br>• Having my boundaries consid-ered
[He] and his brother were in a much more exuberant, 'wild' mood today than last time, they were frolicking, joining in with the exercises and sometimes fooling around. The atmosphere in the exercise room was accordingly high-spirited. [...] the two of them rode the ergometer again for a few minutes, spurring each other on even more.	Participant observation,<br>7-year-old male patient	• Positive experiences regarding social relationships<br>• Having fun<br>• Being able to creatively engage with exercises and adopt ther-apy
"Well, I always enjoy it and I feel really good afterwards."	Interview, 7-year-old male patient	• Having fun<br>• Being happy<br>• Feeling better in my own body
"Yes, the whole perception of the hospital is simply very different, because he can do some-thing like this here. It's easier for him to come here for appointments and we also decided quite spontaneously this morning that we would stay as inpatients today. So we were told that this [exercising] is now possible and then he said, 'Ah well, but then at least I can go to [exercise therapist 1] and [exercise therapist 2] every day, right?'. So it's really a very im-portant topic for him in his head."	Interview,<br>Mother of a 7-year-old male patient	• Distraction / motivation during hospital visits
"Yes, so the joy that he has while [exercising], the glow on his face [...]"	Interview,<br>Father of a 7-year-old male patient	• Being happy<br>• Having fun

3.4 Theme 3: Being recognized and involved as an individual and vulnerable patient<br>**Central organizing concepts: Individualization; Protection; Involvement**		
**Data extract**	**Data source, participant**	**Codes assigned**
"[…] she got the best out of herself without pressure or coercion from anyone present […]"	Interview,<br>Mother of a 6-year-old female patient	• Appreciation of volition
"I've played boules a lot so I have a medal at home and what I really like is this screen ([she] points and moves towards the big FORTEe display) and when we build up all the parkours again."	Interview,<br>6-year-old female patient	• Being able to make my own choices and having variety
"But I find that this personalized [training] in the sense of 'we exercise the way you want, we do the type of sport you feel like', that's another push for me to really enjoy it, because then it's my training and the way I like it."	Interview,<br>16-year-old female patient	• Being able to make my own choices and having variety<br>• Having exercise therapy tailored to my situation<br>• "Being at the center" of therapeutic practice
"So I think it's great that all the people really focus on me; it's super cool with the individual training. Well, we always do what I feel like doing and what I can manage, it's just really tailored and if I don't feel like it or don't have any strength then it's like 'We can also do something more relaxed like for example just a bit of stretching or a bit of acrobatics or something like that'. We just do a lot of strength training […] because I like it so much, and the exercises are really tailored to me"	Interview,<br>16-year-old female patient	• Being able to make my own choices and having variety<br>• Having exercise therapy tailored to my situation<br>• "Being at the center" of therapeutic practice<br>• Having my boundaries considered
"Yes and then in the individual training sessions you can also say 'that's too much for me right now, maybe we can do the next exercise or something'. […] One doesn't have to stick to a plan in such a structured way, but really has the choice of how much you can do now, how much you want to do; and so on."	Interview,<br>16-year-old female patient	• Having my boundaries considered<br>• Having exercise therapy tailored to my situation<br>• "Being at the center" of therapeutic practice

		• Appreciation of volition
"That's why I thought FORTEe was really cool, because I can continue exercising and I'm being supervised and I'm not exposed to any danger."	Interview,<br>16-year-old female patient	• Feeling protected<br>• Being able to continue exercis-ing
"I think it's great that it exists, I think it's great that it's a program, also that it's continuous, also here with the interviews"	Interview,<br>mother of a 10-year-old male patient	• Being involved and asked for my / for our opinion
"Yes, it's just very diverse, you can sometimes even choose what you do"	Interview,<br>10-year-old male patient	• Being able to make my own choices and having variety
"They always ask me, '[The boy's name], do you want to do that?' So, I'm actually allowed to decide more, I tell them to decide, but they say 'No, you can decide what you do.' "	Interview,<br>10-year-old male patient	• Needing guidance and struc-ture; being motivated / prompted to exercise
"No, of course it's especially nice when [his brother] can also take part, that's really, really great when his brother sees how great it is and says 'Wow, […] what you can do here is re-ally cool […]!' "	Interview,<br>father of a 7-year-old male patient	• Having exercise therapy tai-lored to my situation<br>• Positive experiences regarding social relationships

3.5 Domain summary 2: Limitations of exercise therapy		
**Data extract**	**Data source, participant**	**Codes assigned**
"[…] sometimes during dexa[methasone treatment] … exercising didn't work well. […] then I feel like I´m on mute."	Interview, 6-year-old female patient	• Impaired mood and health status create situational limitations / barriers
[…] then I'm just not in the mood [to exercise]. Cortisone makes me feel nothing but hunger."	Interview, 6-year-old female patient	• Impaired mood and health status create situational limitations / barriers
"Sometimes I just don't feel like it, but I think that's normal;"	Interview, 16-year-old female patient	• Impaired mood and health status create situational limitations / barriers
"The chemo also makes you extremely tired, and then you have such a dip."	Interview, 15-year-old female patient	• Impaired mood and health status create situational limitations / barriers
"I'll say three or four weeks ago he was a bit tired and said 'oh no, no exercising' and [the exercise therapists] also came and asked [my son whether he wants to exercise] and [he answered] 'mmm, no.' […] that's when he had a low moment [...]"	Interview, mother of a 10-year-old male patient	• Impaired mood and health status create situational limitations / barriers
"[...] that's the way it is and if he can't or if he just doesn't feel like it then he also says 'ah no mom not today' [...]"	Interview, mother of a 10-year-old male patient	• Impaired mood and health status create situational limitations / barriers to participation
[The exercise therapist] asked [him] if he was ready to exercise, and his hesitant affirmation showed that his mood was not quite so good today. The nurse soon left the room again and now [his] mother greeted us properly. [He] explained that he had pain in his legs today, and his initial agreement became a little less clear again.	Participant observation, 10-year-old male patient	• Impaired mood and health status create situational limitations / barriers to participation
During all these strength exercises, [he] proactively mentioned when he was unable to do something, mostly because he was experiencing pain in his legs. While he was sitting on the	Participant observation, 10-year-old male patient	• Impaired mood and health status create situational limitations / barriers to participation

bench during a short break, I asked him how his legs were feeling, to which he replied that they still hurt.		
At one point […] it was when the possibility of doing pull-ups was brought up—[he], just before attempting them, said that he would rather not do them yet 'because of / with the illness'.	Participant observation, 7-year-old male patient	• Impaired mood and health status create situational limitations / barriers to participation
After the final exercise, stair climbing, [he] sat down on the bench, and suddenly, he seemed unwell. If I remember correctly, [exercise therapist 2] was just in the middle of suggesting that they do another set of the exercise (or was it a different exercise?), but at that moment, he lowered his head and held it in his hands, making it clear that something was wrong. His father and brother immediately turned their attention to him (if I recall correctly, his brother sat down next to him and patted his back).<br>*I wasn't entirely sure at first what exact symptom [he] was experiencing, but I believe he mentioned his 'head', suggesting that it was likely either a headache or dizziness.*	Participant observation, 7-year-old male patient	• Burdensome situations during exercise therapy
"How he just blossomed there, and how he had this joyfulness afterwards, you know? Of course, that only lasts for a while until, at some point, the awareness returns in which phase he's currently in […]."	Interview, father of a 7-year-old male patient	• Limitations to positive psychoemotional effects of exercise therapy
"We had that one incident here where he even fell - we misjudged things a bit. And then, I don't even remember the exact date, but at that time, he was really still very unsteady on his feet."	Interview, father of a 7-year-old male patient	• Impaired mood and health status create situational limitations / barriers to participation
